# A Microfabricated 96-Well 3D Assay Enabling High-Throughput Quantification of Cellular Invasion Capabilities

**DOI:** 10.1038/srep43390

**Published:** 2017-02-27

**Authors:** Rui Hao, Yuanchen Wei, Chaobo Li, Feng Chen, Deyong Chen, Xiaoting Zhao, Shaoliang Luan, Beiyuan Fan, Wei Guo, Junbo Wang, Jian Chen

**Affiliations:** 1 State Key Laboratory of Transducer Technology, Institute of Electronics, Chinese Academy of Sciences, Beijing 100190, P.R. China; 2 Microelectronics Equipment Research and Development Center, Institute of Microelectronics of Chinese Academy of Sciences, Beijing 100029, P.R. China; 3 Department of Vascular Surgery, Clinical Division of Surgery, Chinese PLA General Hospital, Beijing 100853, P.R. China; 4 Department of Cellular and Molecular Biology, Beijing Chest Hospital, Capital Medical University, Beijing 101149, P.R. China

## Abstract

This paper presents a 96-well microfabricated assay to study three-dimensional (3D) invasion of tumor cells. A 3D cluster of tumor cells was first generated within each well by seeding cells onto a micro-patterned surface consisting of a central fibronectin-coated area that promotes cellular attachment, surrounded by a poly ethylene glycol (PEG) coated area that is resistant to cellular attachment. Following the formation of the 3D cell clusters, a 3D collagen extracellular matrix was formed in each well by thermal-triggered gelation. Invasion of the tumor cells into the extracellular matrix was subsequently initiated and monitored. Two modes of cellular infiltration were observed: A549 cells invaded into the extracellular matrix following the surfaces previously coated with PEG molecules in a pseudo-2D manner, while H1299 cells invaded into the extracellular matrix in a truly 3D manner including multiple directions. Based on the processing of 2D microscopic images, a key parameter, namely, equivalent invasion distance (the area of invaded cells divided by the circumference of the initial cell cluster) was obtained to quantify migration capabilities of these two cell types. These results validate the feasibility of the proposed platform, which may function as a high-throughput 3D cellular invasion assay.

Cellular invasion reflects three-dimensional migrations of cells into extracellular matrix, which is central to key physiologic and pathologic activities including white blood cell mediated immune responses and tumor cell mediated metastasis^1^,^2^,^3^,^4^. Assays capable of quantifying cellular invasion capabilities include transwell invasion assays, gel invasion assays, cell exclusion invasion assays, and spheroid invasion assays^5^,^6^,^7^,^8^,^9^.

In a transwell assay, plastic inserts possessing cell-permeable membranes covered with gels composed of extracellular matrix are placed in the wells of a multi-well tissue culture plate, creating two-chamber systems. By placing cells on one side of the gel and a chemoattractant on the other side of the gel, invasion is determined by quantifying the number of cells that traverse the cell-permeable membrane in response to chemical gradients^10^,^11^,^12^. In a gel invasion assay, cells are seeded on top of a gel plug surface and vertical cell migration into the collagen matrix is determined by immunohistochemical staining^13^,^14^,^15^,^16^. These two assays are high throughput and readily available. However, they do not truly mimic the process of 3D cellular invasion since monolayers or even individual cells, rather than cell clusters, are used to initiate the cellular invasion processes.

In a cell exclusion invasion assay, silicone stoppers are initially positioned in individual wells creating exclusion zones. Following cell seeding and spreading, the stoppers are removed and the cells as well as the cell-free circular center regions are overlaid with an extracellular matrix layer, initiating the cellular invasion process^17^,^18^. In a spheroid invasion assay, cell suspensions are loaded into individual wells with round bottom surfaces to form cell spheroids. Following the addition of extracellular matrix, cellular invasions were initiated and monitored by confocal microscopes^19^,^20^,^21^,^22^. These two assays can effectively mimic the 3D invasion of cells from cell clusters. However, they cannot accurately control the geometries and positions of the formed 3D cell clusters, leading to issues of low repeatability and high difficulties in cellular imaging.

Microfabrication is the process of fabricating miniature structures of micrometer scales based on photolithography and due to its dimension comparisons with biological cells, microfabrication is an enabling technique for cellular studies^23^,^24^,^25^,^26^,^27^. More specifically, microfabrication has been widely used to spatially control cellular patterns by regulating the tones of the substrates, producing highly regulated cellular clusters^28^,^29^,^30^,^31^.

This paper further explores the capabilities of microfabrication to construct a 96-well three-dimensional (3D) invasion assay. Compared to conventional 3D invasion assays, the approach proposed in this study can accurately control the geometries and positions of formed 3D cell clusters, significantly improving the device repeatability and throughput. In comparison to previously reported microfabricated approaches of forming controlled 3D cell clusters, in this study, 3D extracellular matrix was formed around the 3D cell clusters to enable characterization of cellular invasion. In addition, the microfabricated setup was designed to be compatible with conventional 96-well plates, which is featured with high throughput and easy access.

## Materials and Methods

### Device Setup and Working Principle

**Figure 1:**
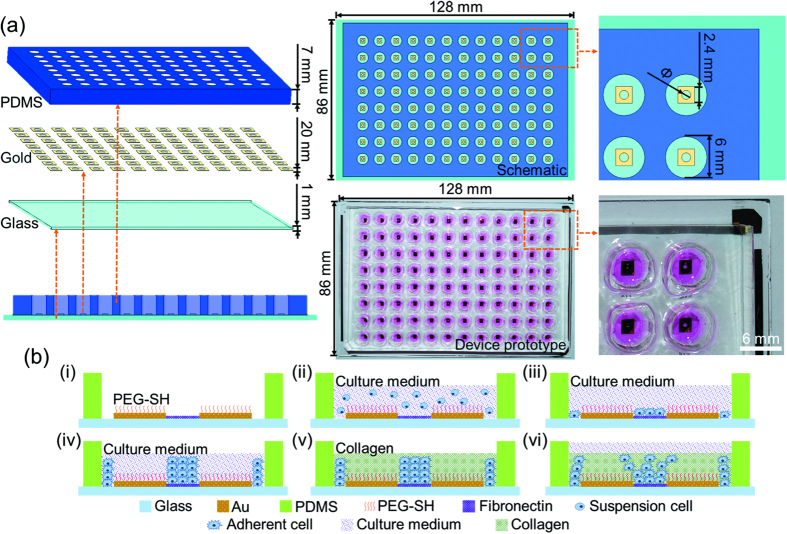
(**a**) The schematic and prototype of 96-well 3D microfabricated cellular invasion assays. The proposed device has has three layers, a glass substrate layer, a layer of micro-patterned gold and a layer of PDMS with through holes to form wells. Within each well, the substrate is divided into two regions, a glass circular region for cell seeding with diameters (Ф) of 200 μm, 400 μm and 800 μm, respectively, as well as a surrounding gold region. (**b**) The device’s working principle. Within each micro well, the gold surface is modified with a self-assembled monolayer of PEG-SH that repels cell adhesion (i). Following cell seeding, cells selectively attach and spread on the fibronectin coated surfaces, forming confluent monolayers (ii,iii). Further cellular proliferation leads to the formation of multilayer cell clusters due to confinement by surrounding PEG molecules (iv). The culture medium in each well is subsequently replaced with collagen solution which forms a 3D invasion matrix upon temperature-induced gelation, thus initiating cell migration (v,vi). Note that in this study, there is a gap between the gold outer boundary and the PDMS inner boundary within each well, which is also used for cell seeding, contributing to the immobilization of 3D temperature-induced matrix. The cellular migrations and invasions in this peripheral areas are not recorded and analyzed.

The 96-well 3D microfabricated cellular invasion assay consists of three layers, a glass substrate layer (a thickness of 1 mm), a layer of micro-patterned gold (a thickness of 20 nm) and a layer of PDMS with through holes to form wells (a thickness of 8 mm) (see Fig. 1(a)). In each well, the substrate is divided into two regions, a glass circular region for cell seeding with diameters (Ф) of 200 μm, 400 μm and 800 μm, respectively, as well as a surrounding gold region at a diameter of 6 mm (see Fig. 1(a)).

The device’s working principle is shown in Fig. 1(b). Within each micro well, the gold surface is modified with a self-assembled monolayer of PEG-SH that repels cell adhesion (i). Following cell seeding, cells selectively attach and spread on the fibronectin coated surfaces, forming confluent monolayers (ii,iii). Further cellular proliferation leads to the formation of multilayer cell clusters due to confinement by surrounding PEG molecules (iv). The culture medium in each well is subsequently replaced with collagen solution which forms a 3D invasion matrix upon temperature-induced gelation, thus initiating cell migration (v,vi).

### Cell Culture

Non-small-cell lung cancer cell lines of A549 and H1299 were cultured at 37 °C in 5% CO_2_ in RPMI 1640 medium supplemented with 10% heat-inactivated fetal bovine serum, 100 units/mL penicillin and 100 μg/mL streptomycin. Immediately prior to an experiment, cells were trypsinized, centrifuged and resuspended in supplemented culture medium for the following experiments. Unless otherwise indicated, all cell-culture reagents were purchased from Life Technologies Corporation (Van Allen Way Carlsbad, CA, USA).

### Device Fabrication

**Figure 2:**
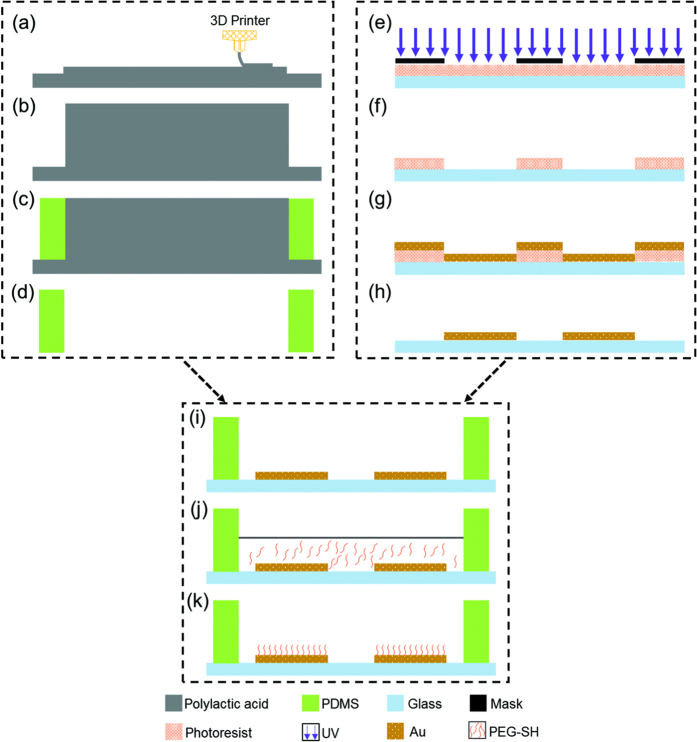
Key fabrication processes include mold master fabrication using 3D printing ((**a**) and (**b**)), PDMS molding ((**c**) and (**d**)), gold pattern formation on glass ((**e**–**h**)), PDMS and glass bonding (**i**), and PEG modification ((**j**) and (**k**)).

Figure 2(a–d) summarizes the key steps of fabricating the PDMS layer with through holes using soft lithography. More specifically, a plastic mold master featured with 96 circular pillars (a height of 15 mm with a diameter of 6 mm) was fabricated by 3D printing (MakerBot Replicator 2X, MakerBot, USA) for PDMS molding (Fig. 2(a) and (b)). PDMS prepolymer and curing agent (184 Silicone Elastomer, Dow Corning Corporation, USA) were then mixed, degassed, poured on the plastic mold master and baked in an oven. After full crosslinking, the PDMS layer was peeled from the master with through holes punched (see Fig. 2(c–d)).

The gold patterns on the glass layer were realized by the lift-off technology, including photoresist exposure (Fig. 2(e), development (Fig. 2(f)), gold deposition (Fig. 2(g)) and photoresist removal (Fig. 2(h)). Following the treatment of oxygen plasma, the PDMS layer and the glass layer with pattern gold features was bonded together to form a 96-well plate (Fig. 2(i)).

Pegylation on the gold surface was realized by soaking the fabricated device in a PEG-SH solution ((11-Mercaptoundecyl)tetra(ethylene glycol), Sigma-Aldrich, USA) for 24 hours (Fig. 2(j) and (k)). Then the device was carefully rinsed in ethanol to remove the residual PEG-SH and to simultaneously sterlize the device for subsequent cell seeding and culture.

### Device Operation and Data Analysis

In this study, A549 and H1299 cells (Non-Small-Cell Human Lung Cancer Cell Lines, China Infrastructure of Cell Line Resources, China) were used as the research models. For A549 cells, cell suspensions at concentrations of 1 × 10^5^, 2 × 10^5^, and 5 × 10^5^ cells/ml were added to fabricated micro wells on day 1 (100 μL per well). For H1299 cells, cell suspensions at 1 × 10^5^ and 2 × 10^5^ cells/ml were added to the micro wells on day 1 (100 μL per well). The microwells featured middle circular regions with a diameter of either 200 μm, 400 μm or 800 μm for cellular seeding. After four days of cell culture and proliferation, cell clusters with multiple layers were formed. Laser scanning microscopy (LSM 700, Zeiss, Germany) was used to characterize the heights of the formed 3D cellular clusters.

Ice-cold collagen solution was added to each well after a four-day cell culture period to form a 3D extracellular matrix around the cell clusters following heat-induced gelation for one hour at 37 °C. Culture medium was then added to the wells. For A549 cells, collagen type I (Rat Tail, Tianjin Weikai Bioeng Ltd., China) was used to form the 3D extracellular matrix and, based on the specification of the company, 20 μL of collagen solution at a concentration of 1 mg/ml was added in each well, followed by the addition of 90 μL culture medium. For H1299 cells, collagen type I (Rat Tail, Corning, USA) was used where 60 μL of collagen solution at a concentration of 2 mg/ml was added in each well based on the specification of the company, followed by the addition of 80 μL culture medium.

After 24 hours of cellular invasion, 2D cellular images were first obtained by a conventional inverted microscope (IX71, Olympus, Japan). Then cells were fixed (Paraformaldehyde, Sigma-Aldrich) and stained (Propidium Iodide, Sigma-Aldrich), with 3D cellular invasion profiles recorded by an upright confocal microscopy (LSM 780, Zeiss, Germany). Image Pro Plus (Media Cybernetics, USA) was used to process the 2D images, producing a key parameter “equivalent invasion distance” (the area of invaded cells divided by the circumference of the initial cell cluster) to quantify the cellular invasion capabilities.

### Statistics

In each group, the measurements of three samples were conducted with results expressed by averages and standard deviations. ANOVA (S-N-K method, coding in Excel) was used for multiple-group comparisons where values of P < 0.05 (*) and P < 0.01 (**) were considered as statistical significance and high statistical significance, respectively.

## Results and Discussion

### Psudo-2D Invasion of A549 Cells

A monolayer of A549 cells formed within the fibronectin-coated circular region one day after cell seeding. Due to the inert nature of the PEG surfaces around the circular regions, A549 cells preferentially proliferated within the circular regions, leading to the formation of 3D cell clusters. After roughly five days of cell culture, PEG based surfaces could not confine the cells within the predefined areas, and thus cell clusters were characterized by laser scanning microscopy after four-days of cell culture.

**Figure 3:**
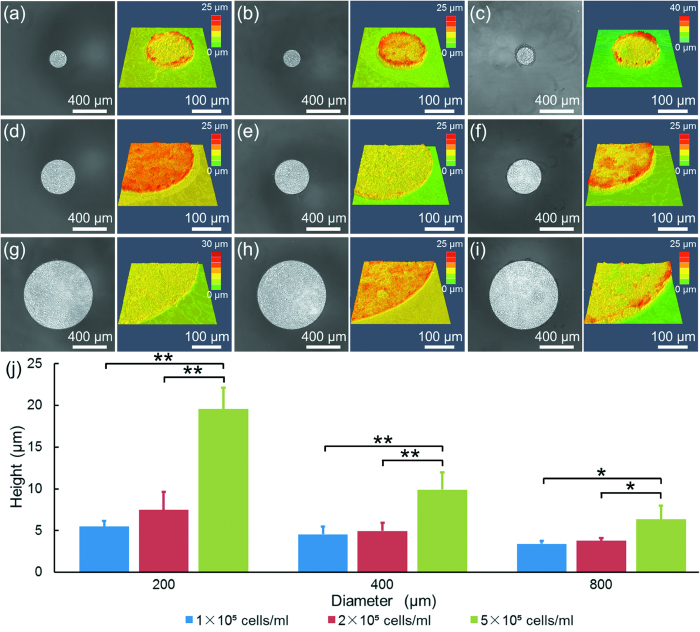
The microscopic and corresponding confocal images of A549 cell clusters as a function of initial diameters and seeding densities at 200 μm and 1 × 10^5^ cells/ml (**a**), 200 μm and 2 × 10^5^ cells/ml (**b**), 200 μm and 5 × 10^5^ cells/ml (**c**), 400 μm and 1 × 10^5^ cells/ml (**d**), 400 μm and 2 × 10^5^ cells/ml (**e**), 400 μm and 5 × 10^5^ cells/ml (**f**), 800 μm and 1 × 10^5^ cells/ml (**g**), 800 μm and 2 × 10^5^ cells/ml (**h**), 800 μm and 5 × 10^5^ cells/ml (**i**). The quantified heights of cell clusters were summarized in (**j**) (*p < 0.05, **p < 0.01).

Figure 3 shows the microscopic and corresponding confocal images of A549 cell clusters after four days of cell culture as a function of initial cellular seeding densities and cluster diameters. Based on image processing, the heights of cell clusters were quantified as 5.5 ± 0.6 μm (seeding density of 1 × 10^5^ cells/ml at the diameter of 200 μm, Fig. 3(a)), 7.5 ± 2.1 μm (seeding density of 2 × 10^5^ cells/ml at a diameter of 200 μm, Fig. 3(b)), 19.6 ± 2.5 μm (seeding density of 5 × 10^5^ cells/ml at a diameter of 200 μm, Fig. 3(c)), 4.5 ± 1.0 μm (seeding density of 1 × 10^5^ cells/ml at a diameter of 400 μm, Fig. 3(d)), 4.9 ± 1.0 μm (seeding density of 2 × 10^5^ cells/ml at a diameter of 400 μm, Fig. 3(e)), 9.9 ± 2.1 μm (seeding density of 5 × 10^5^ cells/ml at a diameter of 400 μm, Fig. 3(f)), 3.4 ± 0.3 μm (seeding density of 1 × 10^5^ cells/ml at a diameter of 800 μm, Fig. 3(g)), 3.8 ± 0.4 μm (seeding density of 2 × 10^5^ cells/ml at a diameter of 800 μm, Fig. 3(h)), 6.3 ± 1.7 μm (seeding density of 5 × 10^5^ cells/ml at a diameter of 800 μm, Fig. 3(i)).

The quantified heights of cell clusters are summarized in Fig. 3(j). Compared to the heights of cell monolayers, which are estimated as roughly 1–2 μm, these height results of A549 cells after four days of cell culture indicated the existence of multiple cell layers or formation of cell clusters. In addition, a trend was observed that a decrease in the diameters of cell clusters and an increase in the cell seeding densities led to an increase in the heights of the cell clusters.

**Figure 4:**
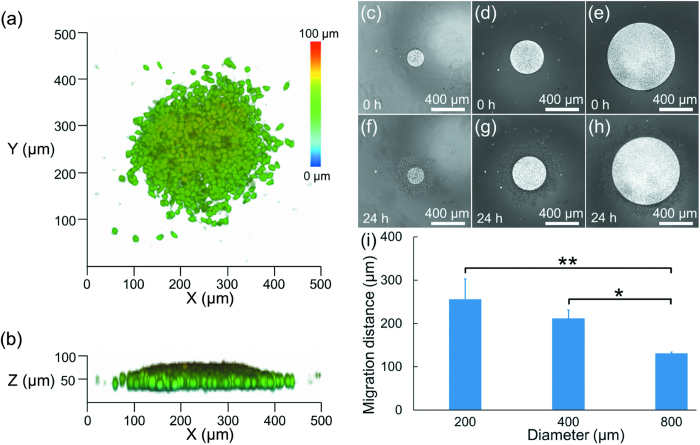
(**a**) and (**b**) 3D reconstruction of the A549 cells invading into the collagen based extracellular matrix for 24 hours. (**c**)–(**e**) 2D microscopic pictures of A549 cell clusters just after the formation of 3D collagen extracellular matrix at the initial diameter of 200 μm (**c**), 400 μm (**d**) and 800 μm (**e**), respectively. (**f**)–(**h**) 2D microscopic pictures of invaded A549 cells 24 hours in the 3D collagen extracellular matrix at the initial diameter of 200 μm (**f**), 400 μm (**g**) and 800 μm (**h**), respectively, with quantified equivalent invasion distances shown in (**i**) (*p < 0.05, **p < 0.01).

In further studies, collagen solution was added to the A549 cell clusters with the initial seeding densities of 5 × 10^5^ cells/ml to initiate cellular invasion (see Fig. 4). Figure 4(a) and (b) show the 3D reconstructions of the A549 cells invading into the collagen based extracellular matrix after 24 hours. These cells were observed to invade into the extracellular matrix following the surfaces previously coated with PEG molecules in a pseudo-2D manner. Figure 4(c–e) are microscopic pictures of A549 cell clusters just after the formation of 3D collagen extracellular matrix while Fig. 4(f–h) show the invaded A549 cells in extracellular matrix after 24 hours.

As shown in Fig. 4(i), for A549 cells, the equivalent invasion distances were quantified as 256.2 ± 46.5 μm (200 μm diameter of the cell cluster), 211.9 ± 19.2 μm (400 μm diameter of the cell cluster), and 130.8 ± 3.3 μm (800 μm diameter of the cell cluster) after 24 hours of cellular invasions. These results indicate that for A549 cells, the increase in the initial cell cluster diameter led to a decrease in the invasion capabilities.

### 3D Invasion of H1299 Cells

**Figure 5:**
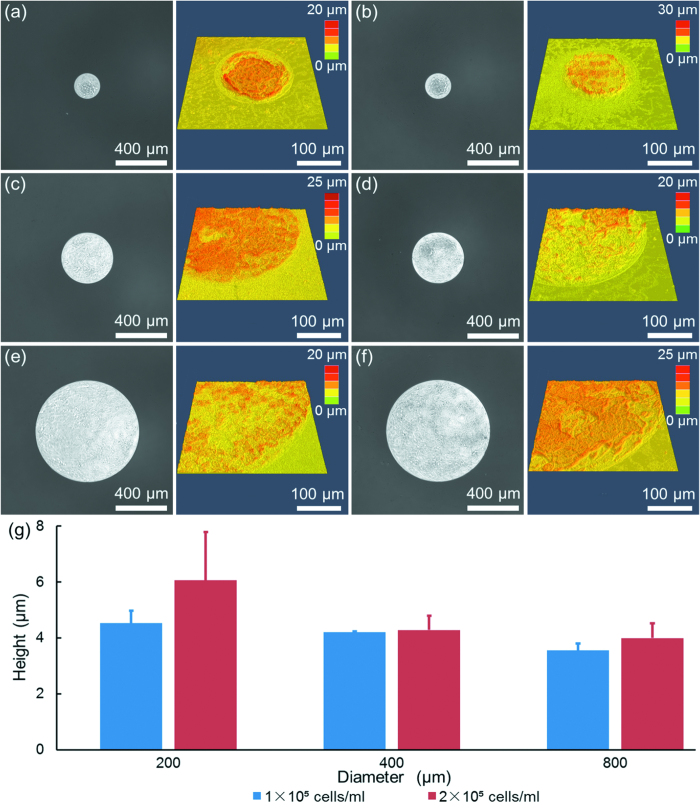
The microscopic and corresponding confocal images of H1299 cell clusters as a function of initial diameters and seeding densities at 200 μm and 1 × 10^5^ cells/ml (**a**), 200 μm and 2 × 10^5^ cells/ml (**b**), 400 μm and 1 × 10^5^ cells/ml (**c**), 400 μm and 2 × 10^5^ cells/ml (**d**), 800 μm and 1 × 10^5^ cells/ml (**e**), 800 μm and 2 × 10^5^ cells/ml (**f**). The quantified heights of cell clusters were summarized in (**g**).

As the second proof-of-concept demonstration, H1299 cells were seeded into the 96-well microfabricated device to evaluate their invasion capabilities. Figure 5 shows the microscopic and corresponding confocal images of H1299 cell clusters after four days of cell culture as a function of initial cellular seeding densities and cluster diameters. The heights of cell clusters were quantified as 4.5 ± 0.5 μm (seeding density of 1 × 10^5^ cells/ml at the diameter of 200 μm, Fig. 5(a)), 6.1 ± 1.7 μm (seeding density of 2 × 10^5^ cells/ml at a diameter of 200 μm, Fig. 5(b)), 4.2 ± 0.1 μm (seeding density of 1 × 10^5^ cells/ml at a diameter of 400 μm, Fig. 5(c)), 4.3 ± 0.5 μm (seeding density of 2 × 10^5^ cells/ml at a diameter of 400 μm, Fig. 5(d)), 3.6 ± 0.2 μm (seeding density of 1 × 10^5^ cells/ml at a diameter of 800 μm, Fig. 5(e)), 4.0 ± 0.5 μm (seeding density of 2 × 10^5^ cells/ml at a diameter of 800 μm, Fig. 5(f)).

The quantified heights of cell clusters were summarized in Fig. 5(g). Again, compared to the heights of monolayers, which are estimated as 1–2 μm, these height results of H1299 confirm the existence of multiple cell layers or the formation of cell clusters. In addition, it was observed that a decrease in the initial diameters of cell clusters and an increase in the initial seeding densities led to an increase in the height of cell clusters, consistent with A549 cell clusters.

**Figure 6:**
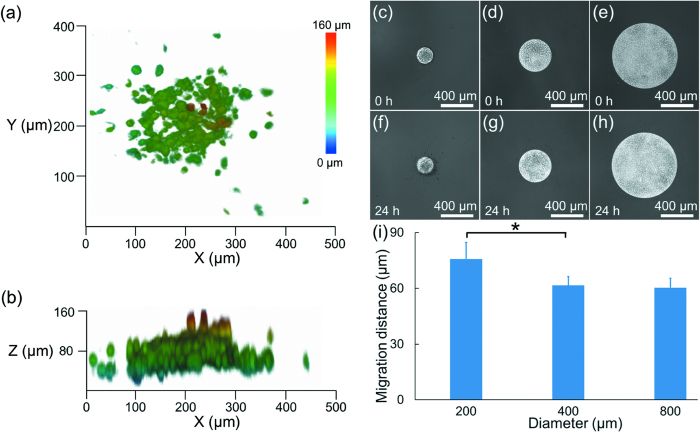
(**a**) and (**b**) 3D reconstruction of the H1299 cells invading into the collagen based extracellular matrix for 24 hours. (**c–e**) 2D microscopic pictures of H1299 cell clusters just after the formation of 3D collagen extracellular matrix at the initial diameter of 200 μm (**c**), 400 μm (**d**) and 800 μm (**e**), respectively. (**f**–**h**) 2D microscopic pictures of invaded H1299 cells 24 hours in the 3D collagen extracellular matrix at the initial diameter of 200 μm (**f**), 400 μm (**g**) and 800 μm (**h**), respectively, with quantified equivalent invasion distances shown in (**i**) (*p < 0.05).

In further studies, collagen solution was added to the H1299 cell clusters with the seeding densities of 2 × 10^5^ cells/ml to initiate the cellular invasion experiments (see Fig. 6). Figure 6(a) and (b) show the 3D reconstructions of the invading H1299 cells, which migrated in a 3D manner including multiple directions rather than migrating along the bottom surface of extracellular matrix as observed for A549 cells. Figure 6(c–e) are microscopic pictures of H1299 cell clusters immediately after the formation of 3D collagen extracellular matrix while Fig. 6(f–h) record the invaded H1299 cells in the extracellular matrix after 24 hours.

As shown in Fig. 6(i), for H1299 cells, the equivalent invasion distances were quantified as 75.8 ± 8.8 μm (200 μm diameter of the cell cluster), 61.5.9 ± 4.8 μm (400 μm diameter of the cell cluster), and 60.4 ± 5.0 μm (800 μm diameter of the cell cluster) after 24 hours of cellular invasion. These results indicated that an increase in the initial H1299 cell cluster diameter led to a decrease in the invasion capabilities of cell clusters, consistent with observations from the A549 cells.

## Conclusions

In this study, a 96-well microfabricated assay was demonstrated to study 3D invasion behaviors of tumor cells. As proof-of-concept demonstrations, the invasion patterns of two different types of tumor cells (A549 and H1299) were observed in which A549 cells invaded into the extracellular matrix following the surface previously coated with PEG molecules in a pseudo-2D manner while H1299 cells invaded into the extracellular matrix in multiple directions as a truly 3D invasion. Future studies may leverage the assay to systematically study and compare invasion capabilities of multiple malignant tumor cells under a variety of conditions including cellular seeding densities, extracellular types and concentrations.

## Additional Information

**How to cite this article:** Hao, R. *et al*. A Microfabricated 96-Well 3D Assay Enabling High-Throughput Quantification of Cellular Invasion Capabilities. *Sci. Rep.* **7**, 43390; doi: 10.1038/srep43390 (2017).

**Publisher's note:** Springer Nature remains neutral with regard to jurisdictional claims in published maps and institutional affiliations.

## References

[b1] Bravo-Cordero J. J., Hodgson L. & Condeelis J. Directed cell invasion and migration during metastasis. Curr Opin Cell Biol. 24, 277–283 (2012). 22209238 10.1016/j.ceb.2011.12.004 PMC3320684

[b2] Friedl P., Locker J., Sahai E. & Segall J. E. Classifying collective cancer cell invasion. Nat Cell Biol. 14, 777–783 (2012). 22854810 10.1038/ncb2548

[b3] Clark A. G. & Vignjevic D. M. Modes of cancer cell invasion and the role of the microenvironment. Curr Opin Cell Biol. 36, 13–22 (2015). 26183445 10.1016/j.ceb.2015.06.004

[b4] Odenthal J., Takes R. & Friedl P. Plasticity of tumor cell invasion: governance by growth factors and cytokines. Carcinogenesis 37, 1117–1128 (2016). 27664164 10.1093/carcin/bgw098

[b5] Valster A. . Cell migration and invasion assays. Methods 37, 208–215 (2005). 16288884 10.1016/j.ymeth.2005.08.001

[b6] Hulkower K. I. & Herber R. L. Cell migration and invasion assays as tools for drug discovery. Pharmaceutics 3, 107–124 (2011). 24310428 10.3390/pharmaceutics3010107 PMC3857040

[b7] Moutasim K. A., Nystrom M. L. & Thomas G. J. Cell migration and invasion assays. Methods Mol Biol. 731, 333–343 (2011). 21516419 10.1007/978-1-61779-080-5_27

[b8] Kramer N. . *In vitro* cell migration and invasion assays. Mutat Res. 752, 10–24 (2013). 22940039 10.1016/j.mrrev.2012.08.001

[b9] Justus C. R., Leffler N., Ruiz-Echevarria M. & Yang L. V. *In vitro* cell migration and invasion assays. J Vis Exp. 88, e51046 (2014). 10.3791/51046 PMC4186330 24962652

[b10] Albini A. . A rapid *in vitro* assay for quantitating the invasive potential of tumor cells. Cancer Res. 47, 3239–3245 (1987). 2438036

[b11] Albini A. & Benelli R. The chemoinvasion assay: a method to assess tumor and endothelial cell invasion and its modulation. Nat Protoc. 2, 504–511 (2007). 17406614 10.1038/nprot.2006.466

[b12] Marshall J. In Cell Migration: Developmental Methods and Protocols (eds Claire Wells M. & Parsons Maddy) 97–110 (Humana Press, 2011).

[b13] Rocha B., Haston W. S. & Freitas A. A. Lymphocyte migration into collagen gels: role of lymph. Scand J Immunol. 19, 297–305 (1984). 6729404 10.1111/j.1365-3083.1984.tb00934.x

[b14] Nystrom M. L. . Development of a quantitative method to analyse tumour cell invasion in organotypic culture. J Pathol. 205, 468–475 (2005). 15685705 10.1002/path.1716

[b15] Timpson P. . Organotypic collagen I assay: a malleable platform to assess cell behaviour in a 3-dimensional context. J Vis Exp. 56, e3089 (2011). 10.3791/3089 PMC3227204 22025017

[b16] McArdle T. J., Ogle B. M. & Noubissi F. K. An *In vitro* inverted vertical invasion assay to avoid manipulation of rare or sensitive cell types. J Cancer 7, 2333–2340 (2016). 27994672 10.7150/jca.15812 PMC5166545

[b17] Lim S. O., Kim H. & Jung G. p53 inhibits tumor cell invasion via the degradation of snail protein in hepatocellular carcinoma. FEBS Lett. 584, 2231–2236 (2010). 20385133 10.1016/j.febslet.2010.04.006

[b18] Glenn H. L., Messner J. & Meldrum D. R. A simple non-perturbing cell migration assay insensitive to proliferation effects. Sci Rep. 6, 31694 (2016). 27535324 10.1038/srep31694 PMC4989229

[b19] Achilli T. M., Meyer J. & Morgan J. R. Advances in the formation, use and understanding of multi-cellular spheroids. Expert Opin Biol Ther. 12, 1347–1360 (2012). 22784238 10.1517/14712598.2012.707181 PMC4295205

[b20] Hirschhaeuser F. . Multicellular tumor spheroids: an underestimated tool is catching up again. J Biotechnol. 148, 3–15 (2010). 20097238 10.1016/j.jbiotec.2010.01.012

[b21] Lin R. Z. & Chang H. Y. Recent advances in three-dimensional multicellular spheroid culture for biomedical research. Biotechnol J. 3, 1172–1184 (2008). 18566957 10.1002/biot.200700228

[b22] Berens E. B., Holy J. M., Riegel A. T. & Wellstein A. A cancer cell spheroid assay to assess invasion in a 3D setting. J Vis Exp. 105, e53409 (2015). 10.3791/53409 PMC4692745 26649463

[b23] Voldman J., Gray M. L. & Schmidt M. A. Microfabrication in biology and medicine. Annu Rev Biomed Eng. 1, 401–425 (1999). 11701495 10.1146/annurev.bioeng.1.1.401

[b24] Folch A. & Toner M. Microengineering of cellular interactions. Annu Rev Biomed Eng. 2, 227–256 (2000). 11701512 10.1146/annurev.bioeng.2.1.227

[b25] Li N., Tourovskaia A. & Folch A. Biology on a chip: microfabrication for studying the behavior of cultured cells. Crit Rev Biomed Eng. 31, 423–488 (2003). 15139302 10.1615/critrevbiomedeng.v31.i56.20 PMC3848900

[b26] Folch A. BioMEMS and cellular biology: perspectives and applications. J Vis Exp. 8, e300 (2007). 10.3791/300 PMC2562485 18989409

[b27] Sackmann E. K., Fulton A. L. & Beebe D. J. The present and future role of microfluidics in biomedical research. Nature 507, 181–189 (2014). 24622198 10.1038/nature13118

[b28] Tan X., Heureaux J. & Liu A. P. Cell spreading area regulates clathrin-coated pit dynamics on micropatterned substrate. Integr Biol. 7, 1033–1043 (2015). 10.1039/c5ib00111k PMC4558397 26205141

[b29] Albert P. J. & Schwarz U. S. Optimizing micropattern geometries for cell shape and migration with genetic algorithms. Integr Biol. 8, 741–750 (2016). 10.1039/c6ib00061d 27334659

[b30] Deglincerti A. . Self-organization of human embryonic stem cells on micropatterns. Nat Protoc. 11, 2223–2232 (2016). 27735934 10.1038/nprot.2016.131 PMC5821517

[b31] Lunova M. . Modulation of collective cell behaviour by geometrical constraints. Integr Biol. 8, 1099–1110 (2016). 10.1039/c6ib00125d 27738682

